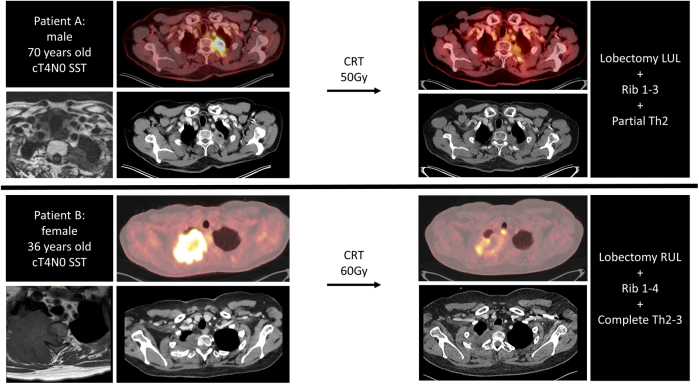# Superior Sulcus Tumors Invading the Spine: Multimodal Treatment Outcomes From the Preimmunotherapy Era

**DOI:** 10.1016/j.jtocrr.2023.100582

**Published:** 2023-10-01

**Authors:** Semih Unal, Ricardo Feller, Agnita Stadhouder, David.J. Heineman, Timothy U. Jiya, Martijn van Dorp, Idris Bahce, Jerry Braun, Suresh Senan, Max Dahele, Chris Dickhoff

**Affiliations:** a Department of Cardiothoracic Surgery, Amsterdam University Medical Center (UMC), Vrije Universiteit Amsterdam, Amsterdam, The Netherlands; b Department of Neurosurgery, Amsterdam UMC, Vrije Universiteit Amsterdam, Amsterdam, The Netherlands; c Department of Orthopedic Surgery, Amsterdam UMC, Vrije Universiteit Amsterdam, Amsterdam, The Netherlands; d Cancer Center Amsterdam, Cancer Treatment and Quality of Life, Amsterdam, The Netherlands; e Orthopedic Clinics Oost Nederland, Hengelo, The Netherlands; f Department of Pulmonary Medicine, Amsterdam UMC, Vrije Universiteit Amsterdam, Amsterdam, The Netherlands; g Department of Cardiothoracic Surgery, Leiden University Medical Center, Leiden, The Netherlands; h Department of Radiation Oncology, Amsterdam UMC, Vrije Universiteit Amsterdam, Amsterdam, The Netherlands

**Keywords:** Superior sulcus, Pancoast tumor, Non–small cell lung cancer, Spine, Pathologic response, Trimodality therapy

## Abstract

**Introduction:**

Curative-intent treatment of superior sulcus tumors (SSTs) of the lung invading the spine presents considerable challenges. We retrospectively studied outcomes in a single center, uniformly staged patient cohort treated with induction concurrent chemoradiotherapy followed by surgical resection (trimodality therapy).

**Methods:**

An institutional surgical database from the period between 2002 and 2021 was accessed to identify SSTs in which the resection included removal of at least part of the vertebral body. All patients were staged using fluorodeoxyglucose positron emission tomography (/computed tomography), computed tomography scan of the chest/upper abdomen, and brain imaging. Surgical morbidity was assessed using the Clavien-Dindo classification. Overall and disease-free survival were calculated using the Kaplan-Meier method.

**Results:**

A total of 18 patients were included: 8 complete and 10 partial vertebrectomies were performed, with six of the eight complete vertebrectomies involving two vertebral levels, resulting in Complete surgical resection (R0) in 94%. Nine patients had a 1-day procedure, and nine were staged over 2 days. The median follow-up was 30 months (interquartile range 11–57). The 90-day postoperative morbidity was 44% (grade III/IV), with no 90-day surgery–related mortality. There were 83% who had a major pathologic response, associated with improved survival (*p* = 0.044). The 5-year overall and disease-free survival were 55% and 40%, respectively. Disease progression occurred in 10 patients, comprising locoregional recurrences in two and distant metastases in eight patients.

**Conclusions:**

Multimodality treatment in selected patients with a superior sulcus tumor invading the spine is safe and results in good survival. Such patients should be referred to expert centers. Future research should focus on improving distant control (e.g. [neo]adjuvant immunotherapy).

## Introduction

For patients with potentially resectable NSCLC located in the superior sulcus, induction chemoradiotherapy (CRT) followed by surgery (trimodality therapy) is a recommended treatment.^1^ This is largely on the basis of the results of the prospective SWOG trial 9416 and Japan Clinical Oncology Group 9806 trials, in which the 5-year overall survival (OS) rates of 54% and 70% were reported, respectively.^2^^,^^3^ However, superior sulcus tumors (SSTs) are frequently not treated according to guidelines; an analysis of the U.S. National Cancer Database revealed that only 25% of patients received neoadjuvant CRT before surgery, negatively impacting the OS.^4^ In the SWOG and Japan Clinical Oncology Group trials, patients were largely clinical stage T3, and despite the complexity of resecting these SSTs, there were cases in which surgery was even more challenging, in particular those involving the spine (T4). This means that, without the necessary multidisciplinary expertise (thoracic, orthopedic, neurosurgery) to resect such tumors, they may be considered technically unresectable or the risks of surgery may be considered too high, resulting in such patients being referred for high-dose CRT alone.^5^ In current practice, this would be followed by durvalumab, with recently reported 5-year OS and disease-free survival (DFS) rates of 42.9% and 33.1%, respectively, for this combination.^6^ Although this is superior to CRT alone, locoregional failure rates are still substantial, which may result in difficult to treat, debilitating pain. To adequately inform the patient and to help multidisciplinary tumor board (MTB) decision-making between trimodality therapy versus CRT plus durvalumab, it is important to know the contemporary results of a trimodality approach for SST with vertebral involvement. We report outcomes for patients who have been uniformly staged and operated on by a surgical team consisting of thoracic, orthopedic, and neurosurgeons.

## Materials and Methods

### Patient Selection and Treatment

This study was approved by the institutional medical ethics committee (approval number 2021.0635). Patients treated with CRT and surgery for SST with vertebral involvement between November 2002 and December 2021 were identified from a surgical database containing 149 patients with SST. SST was defined as lung tumors with radiologic involvement of the thoracic wall above the second rib. Patients with resection of only a transverse process or with metastases at diagnosis were excluded from this analysis. Patients were staged with contrast-enhanced thoracic/upper abdominal computed tomography (CT) scan, whole-body 18F-fluorodeoxyglucose positron emission tomography (18F-FDG-PET) (/CT) scan, brain imaging, and invasive mediastinal when indicated. In patients with possible vertebral involvement, an additional magnetic resonance imaging of the spine was performed to evaluate bone and epidural extension. Patients were restaged according to the eighth edition of the TNM for the current analysis. Resectability was discussed in the MTB attended by thoracic surgeons (with orthopedic and neurosurgeons on invitation) experienced in complex thoracic surgery; the extent of tumor invasion into the vertebral body determined whether the patient was planned for partial or complete vertebral resection. Patients who were considered (borderline) resectable but fit to undergo induction CRT commenced treatment with this strategy. Postoperative morbidity was assessed using the Clavien-Dindo classification.^7^

For patients with squamous cell carcinoma and NSCLC not otherwise specified, chemotherapy typically consisted of one cycle of cisplatin plus gemcitabine before the start of radiotherapy followed by two cycles of cisplatin plus etoposide concurrent with radiotherapy. For patients with adenocarcinoma, three cycles of cisplatin and etoposide with radiotherapy concurrently from day 1 of the first or second cycle of chemotherapy was standard until 2016, when etoposide was replaced by pemetrexed.^8^ Radiotherapy typically comprised 23 or 25 fractions of 2 Gy in upfront resectable tumors and 30 or 33 fractions of 2 Gy when there were any doubts about resectability. In the absence of disease progression on whole-body fluorodeoxyglucose 18F–FDG-PET and CT scan approximately 3 weeks after the last fraction of radiotherapy, and after repeat MTB discussion, patients were scheduled for surgery approximately 6 weeks after the last day of radiotherapy.^9^ All surgeries were performed by a team of thoracic, orthopedic, and neurosurgeons in a single tertiary referral center, in which approximately nine patients/year with SST are operated on, making it the highest volume center for SST surgery in the Netherlands.^10^ Details on the surgical procedure can be found in Supplementary Data 1.

On histologic examination, pathologic complete response (pCR) was defined as the absence of viable tumor cells, and major pathologic response (MPR) as less than or equal to 10% residual viable tumor cells within the primary tumor bed. Consistent with national guidelines, follow-up CT scans were planned every 3 months in the first 2 years after surgery, every 6 months in years 2 to 5, and yearly thereafter.^11^ Missing data were obtained by contacting the referring physician or general practitioner.

### Statistics and Outcome

DFS was defined as the time between surgery and date of (locoregional or distant) recurrence, or death in the absence of disease progression. OS was defined as the time between surgery and the date of death of any cause or last follow-up (May 1, 2022). Locoregional recurrence was defined as objective tumor progression or relapse in the area of previous surgery or locoregional lymph nodes. Normally distributed continuous variables were presented as means and SD, non-normally distributed variables by their median and interquartile range (IQR) or 95% confidence interval (CI). Normally distributed continuous data was tested with the independent samples Student’s *t* test. Non-normally distributed data was tested with the Mann-Whitney *U* test. Categorical variables were presented as frequencies with percentages and tested using Pearson’s chi-square test or Fisher’s exact test, as appropriate. The Kaplan-Meier method was used to test survival. When available the confidence intervals of the median are presented in the figure. All analyses were conducted using the Statistical Package for the Social Sciences software version 26.0 (IBM SPSS Statistics, IBM Corp., New York).

## Results

Of the 149 patients operated on from 2002 to 2022 for SST, 18 (13 men and five women) met the study inclusion criteria. The mean patient age was 53.4 years (SD ± 11.8). Demographics and clinical characteristics are summarized in Table 1. Pretreatment tumor histologic diagnosis was adenocarcinoma (n = 8), squamous cell carcinoma (n = 5), and NSCLC not otherwise specified (n = 5). All patients, except one, completed planned CRT. Concurrent radiotherapy doses were 46 or 50 Gy (n = 7), and 60 or 66 Gy (n = 11) (*p* = 0.88). Before resection, all patients were restaged with a PET-CT. Examples of pre-CRT and post-CRT CT, PET-CT, and magnetic resonance imaging images of patients with partial vertebrectomy or a complete vertebrectomy are presented in Supplementary Data 2.

**Table 1:** Demographics and Clinical Characteristics of Patients With Superior Sulcus Tumors and Vertebral Involvement, Treated With Chemoradiotherapy and Surgery Between 2002 and 2022

Clinical Characteristics	n	%
Patient and tumor characteristics		
Number of patients	18	
Sex (male:female)	13 vs. 5	
Mean age (SD)	53 (11.8)	
ASA classification		
2	8	44
3	10	56
ECOG performance status		
0	17	94
1	1	6
T status on imaging		
T4	18	100
Invasive mediastinal evaluation		
EUS	4	22
EBUS	4	22
Mediastinoscopy	2	11
Clinical Nodal status		
No lymph nodes	17	94
Hilar	1	6
Histopathology		
NSLC-NOS	5	28
Adenocarcinoma	8	44
Squamous cell carcinoma	5	28
Laterality		
Right	13	72
Left	5	28
Treatment characteristics		
Radiotherapy, planned dose		
46 or 50 Gy	7	39
60 or 66 Gy	11	61
Dose reduction radiotherapy		
0%	17	94
25%	1	6
Surgery performed in the study period		
2002–2012	6	33
2013–2022	12	67
Surgical approach (thoracic part)		
Anterior	2	11
Posterolateral	15	83
Combined	1	6
Type of pulmonary resection		
Lobectomy	17	94
Lobectomy and wedge	1	6
Resection thoracic wall		
Two ribs	1	6
Three ribs	9	50
Four ribs	7	39
Five ribs	1	6
Type of vertebral resections and levels		
Complete		
T 2		
T 1–2	1	6
T 2–3	2	11
T 2–4	4	22
Partial	1	6
T 1	1	6
T 2	1	6
T 1–2	1	6
T 1–3	1	6
T 2–3	4	22
T 3–4	1	6
C7–T1	1	6
Resection margin		
Complete resection (R0)	17	94
Microscopically incomplete (R1)	1	6
Outcome		
90-day surgical morbidity (Clavien-Dindo classification)		
Grade 3a	3	17
Grade 3b	3	17
Grade 4	2	11
ypTNM (eighth edition 2017–2019)		
0	7	39
Ia	3	17
Ib	0	0
IIa	1	5
IIb	2	11
IIIa	5	28
Total in-hospital stay, days (median, IQR)		
Surgical ward	13 (10–22)	
Intensive care unit	1.5 (1–6)	

ASA, American Society of Anesthesiologists; ECOG, Eastern Cooperative Oncology Group, EUS, endoscopic ultrasound; EBUS, endobronchial ultrasound; NOS, not otherwise specified; yp, post-neoadjuvant pathologic stage; IQR, interquartile range.

Anatomical pulmonary resection with en bloc resection of the thoracic wall and vertebra was performed by means of posterolateral thoracotomy (n = 15), anterior thoracotomy (n = 2), or combined approach (n = 1). Surgery was either planned as a 1-day (partial vertebrectomy n = 8, complete vertebrectomy n = 1) or a 2-day procedure (partial vertebrectomy n = 2, complete vertebrectomy n = 7). Complete vertebrectomy was performed at level T2 (n = 1), T1 plus 2 (n = 2), T2 plus 3 (n = 4) and T2 to 4 (n = 1). The median total operating time was 594 minutes (IQR: 333–884) for partial vertebrectomy and 929 minutes (IQR: 775–1098) for complete vertebrectomy. The 90-day postoperative morbidity was 44% (Clavien-Dindo grade III n = 6, grade IV n = 2). During follow-up, six patients needed one or more re-operations because of progressive cervical-thoracic kyphosis (n = 3: 2 partial vertebrectomy, one complete vertebrectomy), deep infection (n = 2, both complete vertebrectomy) and painful cutaneous pressure of a dorsal facet-screw (n = 1: complete vertebrectomy). Complete surgical resection (R0) was achieved in 17 patients (94%). An MPR was found in 15 patients (83%), of which seven had pCR (39%).

### Outcome

Follow-up was complete for all patients up to May 1, 2022. The median follow-up was 30 months (IQR: 11–57). At the end of follow-up, 11 of 18 patients (61%) were alive, of which 4 (36%) patients had disease recurrence. Six out of seven deaths (86%) were disease related. The 90-day postoperative mortality rate was 11% (n = 2), both after partial vertebrectomy, although the cause of death was not related to the operation—one patient died of a ruptured abdominal aneurysm, and another died of complications from a cardiac metastasis, which was confirmed at autopsy. The median hospital stay was 13 days (IQR: 10–22) including a median of 1.5 days in the intensive care unit (ICU) (IQR: 1 – 6).

**Figure 1:**
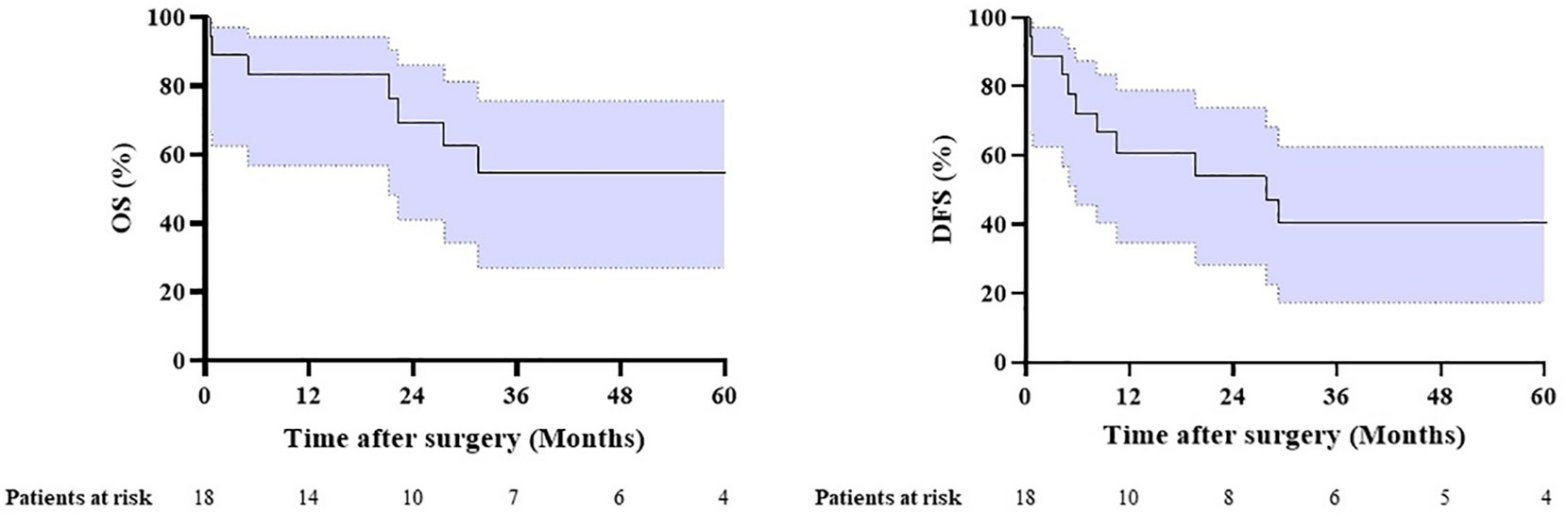
OS and DFS for patients with SST and vertebral involvement treated with chemoradiotherapy and surgery. OS, overall survival, DFS, disease-free survival, SST, superior sulcus tumor.

**Figure 2:**
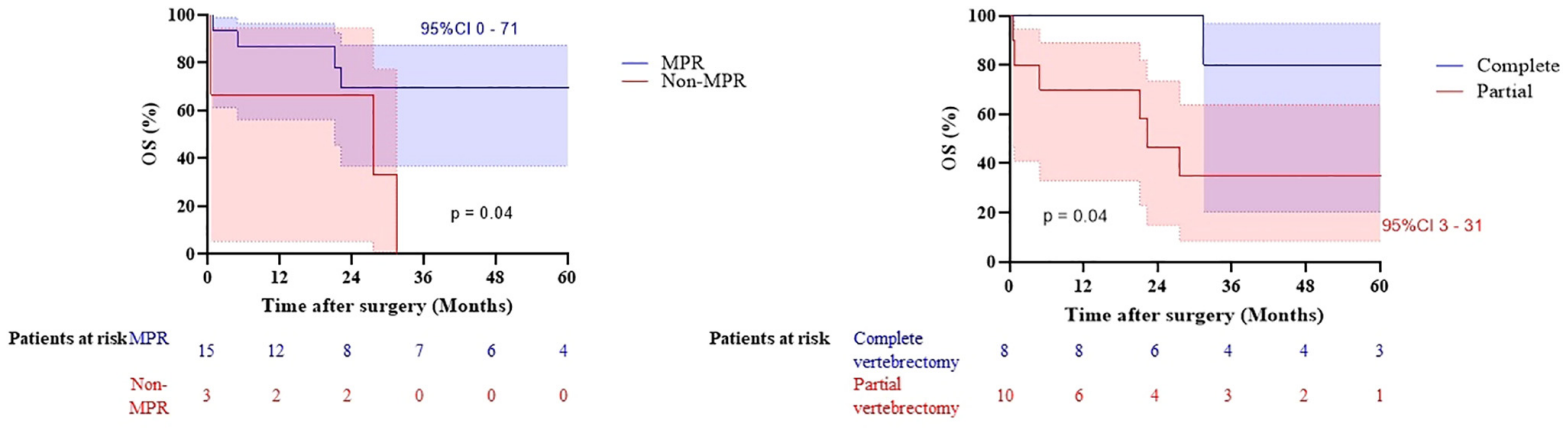
Survival curves of patients with SST invading the spine and major pathologic response versus nonmajor pathologic response and of patients with a partial compared with a complete vertebrectomy. CI, confidence interval; MPR, major pathologic response; OS, overall survival; SST, superior sulcus tumor.

The median DFS was 28 months (CI: 5–50) with 2- and 5-year DFS rates of 54% and 40%, respectively (Fig. 1). Recurrence of disease was diagnosed in 10 patients (56%); locoregional recurrence (n = 2), and distant metastases (n = 8) located in the brain (n = 4), lung (n = 1), brain and bone (n = 1), adrenal glands and lung (n = 1), and adrenal glands and bone (n = 1). The OS at 2- and 5-years was 69% and 55%, respectively (Fig. 1). The median OS was not reached. Although the numbers were small, patients with MPR (n = 15) had a significantly improved 5-year survival when compared with those patients with non-MPR (n = 3): 69% versus 0%, respectively (*p* = 0.044) (Fig. 2).

Complete vertebrectomies were more often performed in a 2-day procedure (n = 7 [87.5%] versus n = 2 [20.0%], *p* = 0.02) (Table 2). Complete vertebrectomy was associated with better survival than partial vertebrectomy (*p* = 0.040) (Fig. 2). During follow-up, six patients with a partial vertebrectomy had died, of which 5/6 (83%) were because of disease progression. In the complete vertebrectomy group, one patient (12.5%) died with the progression of the disease. The median OS for partial vertebrectomy was 22 months (CI: 13–31) with a median of 6 months (CI: 1–11) for DFS. In patients with a complete vertebrectomy, the median for OS was not reached and DFS at 2 and 5 years was 88% and 58%, respectively. Disease recurrence was more frequent in partial vertebrectomy (70%) (locoregional [n=1], distant [n = 6]) when compared with complete vertebrectomy (37.5%) (locoregional [n=1] and distant [n = 2]).

**Table 2:** Comparison of Characteristics Between Partial (n = 10) and Complete (n = 8) Vertebral Resections

Clinical Characteristics and Outcome	Partial (n = 10)	Complete (n = 8)	*p* Value
Y of resection			0.15
2002–2012	5	1	
2013–2020	5	7	
Radiotherapy dose			0.37
<60 Gy	5	2	
≥60 Gy	5	6	
Surgery			0.02
1-day procedure	8	1	
2-day staged procedure	2	7	
Resection margin			0.44
R0	10	7	
R1	0	1	
Pathologic response			>0.99 (MPR vs. non-MPR)
Non-MPR	2	1	
MPR (pCR)	5 (3)	3 (4)	
Hospital stay in d, median (IQR)	12 (10–22)	15 (11–36)	0.32
Intensive care unit stay in d, median (IQR)	1 (0–5)	4 (1–6)	0.15
Follow-up in mo, median (IQR)	22 (4–41)	41 (23–81)	0.10
Recurrence of disease	7 (70%)	3 (37.5%)	0.34
Local	1	1	
Distant	6	2	
Overall survival			0.04
2-y	47	80	
5-y	35	80	
Disease-free survival			0.02
2-y	27	88	
5-y	27	58	

IQR, interquartile range; MPR, major pathologic response; pCR, pathologic complete response.

The median hospital stay for patients with partial vertebrectomy was 12 days (IQR: 10–22), of which 1 day (IQR: 0–5) was in the ICU. In patients with complete vertebrectomy, the median hospital stay was 15 (IQR: 11–36) days of which 4 days (1–6) were spent in the ICU. Table 2 presents the outcome of patients with partial vertebrectomy compared with those with complete vertebrectomy.

## Discussion

In this series of patients with SST invading the spine, treatment with induction CRT followed by surgery resulted in 5-year OS and DFS rates of 55% and 40%, respectively, with acceptable 90-day perioperative surgical morbidity and no surgical mortality. Although this is not the largest series to date, it is homogenous in terms of preoperative staging with PET-CT and induction treatment with CRT. The literature mostly consists of case reviews or smaller cohort studies and accurate interpretation of the published data is hampered by varying definitions of vertebral involvement, with some authors considering patients with involvement in the transverse process, and its subsequent resection, as a partial vertebral resection.^12^, ^13^, ^14^ This is in contrast with our series, in which only true partial or complete vertebral body resections were included and analyzed. Despite this strict definition, the outcomes were comparable to those in the available literature. A systematic review reporting on 135 patients with SST invading the spine reported a 5-year survival rate of 43%, and a large French series by Collaud et al.,^14^^,^^15^ in which 48 patients were included, reported a 5-year OS rate of 61%.

Several factors may have contributed to the good outcomes in our series. First is patient selection: all patients were uniformly staged, including PET-CT scan and brain imaging before the start of induction treatment. Second is the expertise of the surgical team: this includes orthopedic surgeons and spinal neurosurgeons and, in addition, a large team, familiar with complex thoracic oncology patients, such as anesthesiologists, nurses (including ICU), physiotherapists, and dieticians. In addition, the thoracic surgical team has developed specific experience in complex thoracic surgery and performs the most SST resections annually in the Netherlands.^10^ Third is practice evolution: over the years, we have increasingly preferred the 2-day procedure over the 1-day procedure, which is in line with Collaud et al.^15^ in their series of 48 patients, of which 23 (48%) were treated in this way. In our experience, 1-day surgical sessions were long and went on until late in the evening, which we believe is unfavorable for pulmonary vitality and pressure ulcers and is more intense for the surgical team. A possible explanation for better outcomes after two-stage procedures could be that there is less patient stress, less depression of the immune system, and less induction of circulating tumor cells, which may have led to a lesser extent or occurrence of distant progression.^16^^,^^17^ Furthermore, an in-between overnight stay in the ICU or Post Anesthesia Care Unit facilitates pulmonary, physiological, and physical recovery of the patient, and destresses the surgical team. And fourth is the radiotherapy dose: the median dose in the SWOG study was 45 Gy, whereas in our study, patients received higher doses, with the majority having at least 60 Gy.^2^ In addition, radiotherapy delivery techniques and dose distributions have improved over the years, facilitating organ-at-risk sparing and allowing higher tumor doses to be delivered closer to the dose-limiting spinal cord, resulting in high pathologic response rates facilitating radical resection and resulting in improved OS.^18^ This hypothesis is supported by the results of a recent study reporting good results of extended resections after induction treatment in T3/T4 NSCLC.^19^

An unexpected finding was the improved survival of patients with complete versus partial vertebrectomy, most likely attributable to a higher rate of disease recurrence in partial vertebrectomies (70.0% versus 37.5%). As R0, MPR rates, and radiotherapy doses were comparable, possible explanations for this finding are that complete vertebrectomies were mainly (88%) performed in the second part of the study (2013-2020) in which the 2-day procedure was increasingly adopted as common practice (see comments above). Other factors, such as better selection of patients by the MTB, and increasing experience of the surgical team in the 2013 to 2020 period, may also have had an impact on outcomes. However, the numbers were too small to perform multivariate testing.

Distant metastases are still of major concern and mostly occur in the brain within 3 years after surgery. Several studies have identified MPR and pCR as predictive factors for improved survival, and although the numbers were small, our study confirmed these findings. Treatments that improve pathologic response rates are likely to have an impact on DFS and OS by reducing locoregional and distant recurrence. Several studies are now investigating immunotherapy combined with CRT in the neoadjuvant setting. Although definitive data are awaited, initial results are promising.^20^ Adding immunotherapy to CRT in the consolidative setting has been found to improve DFS and OS in patients treated for unresectable stage III NSCLC, with 5-year OS and DFS rates of 42.9% (CI 38.2 to 47.4) and 33.1% (28.0 to 38.2), respectively.^5^ Whether this beneficial effect could be extrapolated to the addition of adjuvant immunotherapy after trimodality therapy for SST, including patients with spine involvement, is currently unknown; but it merits further investigation as a means of improving local and distant control rates while avoiding the potential for increased surgical difficulties when adding immunotherapy to CRT in the neoadjuvant setting.^13^ The results of our study reveal that, even in patients requiring lung, chest wall, and complete (single/multilevel) vertebral resection, trimodality therapy alone, without any adjuvant immunotherapy, is safe and delivers high rates of 5-year OS and local control. When placed side-by-side with the results of CRT and durvalumab for unresectable disease, they compare very favorably. We acknowledge that the results from this study may not be reproducible in other centers, as the surgery was performed by an experienced multidisciplinary team. In centers without access to this type of surgery, we encourage referral of the kinds of patients described here to an appropriately experienced MTB.

In conclusion, for patients with SST and vertebral involvement, concurrent CRT followed by surgical resection resulted in a 5-year OS and DFS rate of 55% and 40%, respectively. These patients should be discussed in an MTB attended by surgeons with experience in complex thoracic surgery. Distant control remains a major concern but it is hoped that it can be improved with the addition of immunotherapy in the neoadjuvant or adjuvant setting. This is currently being investigated in several ongoing trials.

## CRediT Authorship Contribution Statement

**Semih Unal:** Conceptualization, Data curation, Formal analysis, Investigation, Methodology, Resources, Software, Visualization, Roles/writing - original draft.

**Ricardo Feller:** Data curation, Resources, Roles/writing - original draft.

**Agnita Stadhouder:** Data curation, Resources, Roles/writing - original draft

**David. J. Heineman:** Roles/writing - original draft, Writing - review & editing.

**Idris Bahce:** Writing - review & editing.

**Timothy U. Jiya:** Writing - review & editing.

**Martijn van Dorp:** Formal analysis, Methodology, Software, Visualization, Roles/writing - original draft.

**Jerry Braun:** Writing - review & editing.

**Suresh Senan:** Conceptualization, Writing - review & editing.

**Max Dahele:** Conceptualization, Formal analysis, Methodology, Resources, Software, Supervision, Visualization, Roles/writing - original draft, Writing - review & editing.

**Chris Dickhoff:** Conceptualization, Data curation, Formal analysis, Investigation, Methodology, Project administration, Resources, Supervision, Visualization, Roles/Writing - original draft, Writing - review & editing.

## Declaration of Generative AI and AI-assisted technologies in the writing process

The authors disclose the usage of generative AI and AI-assisted technologies in the writing process.

## Figures and Tables

**Supplementary Figure 1:**
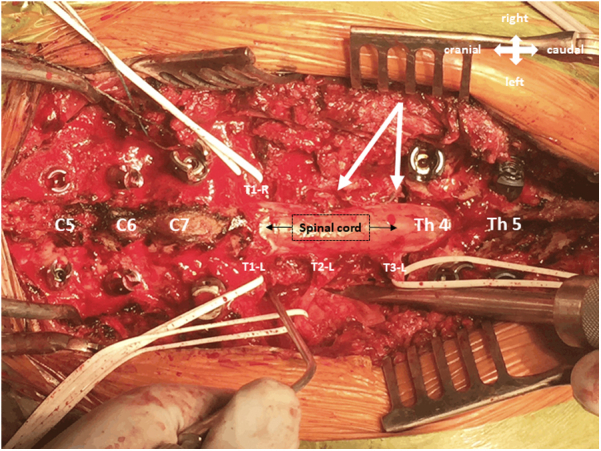


**Supplementary Figure 2:**
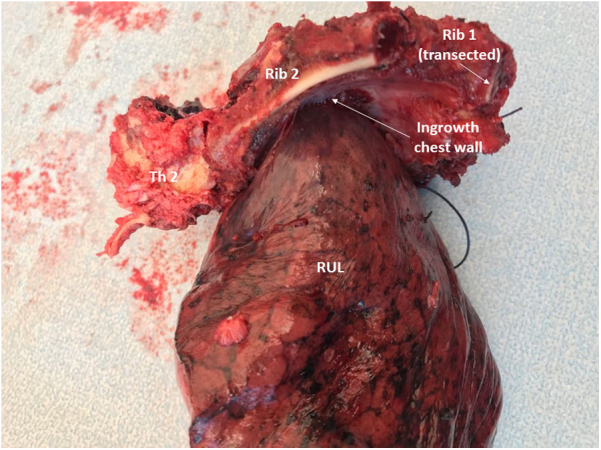


**Supplementary Figure 3:**
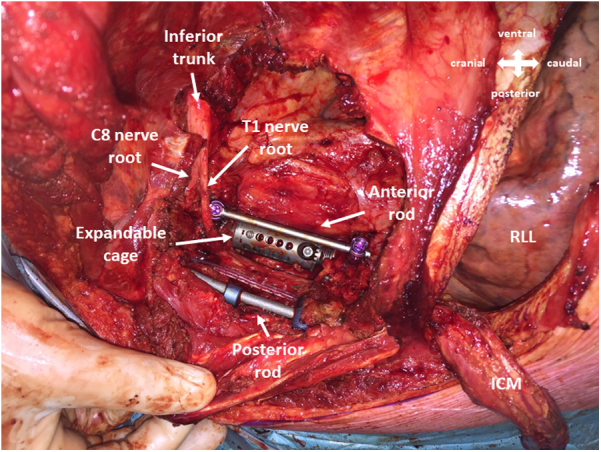


**Supplementary Figure 4:**