# Supplementary material for: Superior Sulcus Tumors Invading the Spine: Multimodal Treatment Outcomes From the Preimmunotherapy Era
Source: JTO Clin Res Rep. 2023 Oct 1;4(12):100582. doi: 10.1016/j.jtocrr.2023.100582 (PMC10689281; doi:10.1016/j.jtocrr.2023.100582)
Supplement: Supplementary File [file mmc1.docx]

**Supplementary file S1**

*Surgical procedure*

Anatomical pulmonary resection of the lung was standard, with en-bloc resection of the thoracic wall and involved vertebra(e). Patients were planned as either a 1-day procedure, or a 2-day staged procedure in which the patient stayed overnight on the intensive care unit (ICU) or post-anaesthesia care-unit (PACU). Here, we briefly describe the 2-day procedure for complete vertebral resection.

On the first day, surgery is performed in the prone position with a Mayfield frame for cervical stabilization. The objective of the first day is to perform a stabilization of the spine with mobilization of the segment to be resected the next day from anterior. Through a posterior midline incision, pedicle screws and/or facet screws are placed, usually two to three segments cranial and caudal from the affected spinal segment. Laminectomy is then performed at the involved spinal segment, taking care not to breach the facet joints at the affected side whilst extending to remove the facet, costo-transverse and the cost-vertebral joints at the non-affected side. The cranial and caudal adjacent intervertebral discs of the involved spinal segment are dissected and then the involved vertebral segment is mobilised as far anteriorly as possible, at the non-affected side. Nerve roots are ligated and cut when necessary. Posterior spinal instrumentation is finally completed using two titanium rods and a transverse connector (Figure 2). After stabilization is completed and the wound closed, the patient is transferred to the ICU/PACU, with the released part of the spine in situ, to be removed the next day. Patients were not mechanically ventilated overnight, but awake and conscious between the surgeries.

On the second day, the patient is placed in a lateral decubitus position and the trapezius and rhomboid muscles are cut through a posterolateral incision allowing mobilisation of the scapula to adequately expose the chest wall and the thoracic outlet. An anatomical resection of the lung is then performed, and once hilar structures are transected, the involved ribs are cut. Structures passing the thoracic outlet (subclavian vein and artery, brachial plexus), are mobilised away from the first rib. The nerve root of Th1 is transected when involved by the tumor. Dissection and mobilization of the spinal segment is completed. Finally, the lung, thoracic wall and the affected vertebrae are removed from the thoracic cavity with a rotational manoeuvre around the spinal cord (Figure 3).

In complete vertebrectomies, the spinal segment is reconstructed with an expandable cage and when feasible, augmented with anterior instrumentation (Figure 4). Finally, lymph nodes are removed, the bronchial stump is buttressed with an intercostal muscle flap, and chest tubes are placed. Patients are then extubated and monitored on the PACU for one night before being transferred to the surgical ward

**Figure legends:**

*Supplementary figure 1:*

Patient with a superior sulcus tumor invading the spine over two levels: patient in supine position: laminectomy Th1(partial)-Th2-Th3-Th4(partial); nerve roots T2 and T3 transected on the right side (arrows); nerve roots T1 (T1-R) on the right side and T1-3 (T1-L, T2-L, T3-L) on the left are encircled with vessel loops; facet-screws in C5-C6, pedicle-screws in Th1 and Th4-6.

*Supplementary figure 2***:** Resection specimen of a tumor originating from the right upper lobe (RUL) invading the spine at level Th2. The vertebral body of Th2 is resected en-bloc with ribs 1-2 and the RUL.

*Supplementary figure 3:* A patient in whom two vertebral bodies are replaced (Th1-Th2) by an expandable cage. The posterior rod (inserted day one) is visible in the operative field after removal of the specimen. Here, the T1 nerve root is spared; it forms the inferior trunk of the brachial plexus with the nerve C8. The intercostal muscle flap (ICM) is used to buttress the bronchial stump (RLL = right lower lobe).

**Supplementary file S2:**

*Pre- and post chemoradiotherapy CT, PET-CT and MRI-images of a patient with partial vertebrectomy (A) and a patient with complete vertebrectomy (B).*

**Figure legend:**

*Supplementary figure 4:*

Pre- and post chemoradiotherapy imaging of two patients with a superior sulcus tumor invading the spine. Patient A was a 70 years old male with a superior sulcus tumor protruding into the neuroforamen of T2, and with cortical invasion of the body of the second thoracic vertebra (Th2). Surgery followed induction therapy with CRT up to 50Gy. Patient B was a 36 years old female, with a large superior sulcus tumor with vertebral corpus invasion of level Th2 and Th3, and growing into the spinal canal. This tumor was considered borderline resectable upfront, so induction radiotherapy was increased to a radical dose of 60Gy

(Abbreviations: RUL = right upper lobe; Th2 = Second thoracic vertebra; CRT = chemoradiotherapy; SST = superior sulcus tumor; LUL = left upper lobe; Th2-3 = second and third thoracic vertebra)
